# Initiation and maintenance of lifestyle changes among participants in a healthy life centre: a qualitative study

**DOI:** 10.1186/s12889-020-09111-8

**Published:** 2020-06-26

**Authors:** Cille H. Sevild, Christopher P. Niemiec, Lars Edvin Bru, Sindre M. Dyrstad, Anne Marie Lunde Husebø

**Affiliations:** 1 Department of Public Health, University of Stavanger, Stavanger, Norway; 2 Center for Health Promotion, Research Unit, Stavanger, Norway; 3 Department of Psychology, University of Rochester, Rochester, USA; 4 Centre for Learning Environment, University of Stavanger, Stavanger, Norway

**Keywords:** Emotional coping, Healthy life centre, Lifestyle change, Motivation, Qualitative, Self-determination theory, Self-regulation skills

## Abstract

**Background:**

Since the early 2000s, Healthy Life Centres have been established in Norway to promote physical and mental health. Yet to date, little is known about the efficacy of Healthy Life Centres in promoting health behaviour change and maintenance or the factors that underlie these processes. Accordingly, the aim of the current study was to examine the factors that participants in a Healthy Life Centre perceive as relevant for the initiation and maintenance of lifestyle changes toward more physical activity and consumption of a healthier diet.

**Method:**

Participants were purposely recruited from among adherers in a 12-month multi-method research project at a Healthy Life Centre. Individual, semi-structured interviews were conducted with 8 women and 6 men who were between the ages of 20 and 61 years old. Data were analysed using Systematic Text Condensation.

**Results:**

Three main themes were derived from this analysis. The first theme focused on the motives behind initiation and maintenance of lifestyle changes along with the importance of a relationally supportive environment to promote perceived competence in pursuing a healthy lifestyle. The second theme focused on strategies for coping with the challenges and potential pitfalls that were associated with various unpleasant experiences and life events. The third theme focused on several specific skills that were helpful to the initiation and maintenance of lifestyle changes.

**Conclusion:**

The current study enhanced an understanding of the initiation and maintenance of lifestyle changes, although these processes were not disentangled in participants’ experiences. In line with self-determination theory, the results suggested that lifestyle change is more likely to be initiated and maintained when goals are not only achievable but also regulated with autonomous motivation and of intrinsic value. Conversely, lifestyle change is difficult to maintain when motives are external to the self. Further, cognitive and behavioural skills were valuable and necessary in coping with unpleasant emotions. Finally, the critical function of self-regulation skills for making realistic plans and prioritizations in order to balance healthy lifestyle behaviours with the routines of “daily life” while monitoring outcomes was readily apparent. Healthy Life Centres can contribute to these processes in meaningful ways.

## Background

On average each year, 71% of all mortalities globally occur from non-communicable diseases (NCDs), and the risk of NCDs is increased by lifestyle behaviours such as physical inactivity and unhealthy diet [1, 2]. Indeed, the percentage of individuals in high-income Western countries who do *not* meet their recommended level for physical activity is alarmingly high [3, 4], and health-depleting diets are consumed around the world [5]. These risk factors for NCDs can be lowered through modification of lifestyle behaviours [2]. Yet these modifications are remarkably difficult to achieve, as previous research on maintenance of treatment gains around physical activity and diet is limited [6] and equivocal [7].

Since the early 2000s, the Norwegian Directorate of Health has recommended establishing Healthy Life Centres (HLCs) as a part of primary health care services in Norway. The main goal of the HLCs is to promote physical and mental health through structured, individual and group experiences that focus on physical activity and diet [8]. Yet to date, little is known about whether or not HLCs can successfully promote behaviour change and maintenance. For instance, one study revealed that participants in HLCs did not report an increased level of physical activity [9]. A second study, with a high rate of dropout, revealed that participants who adhered to the program reported an improvement in general health [10]. A third study revealed that participants in HLCs had a reduced risk of diabetes after 24 months [11]. Other studies that examined the initiation of health behaviour change found that emotional baggage and feeling “stuck” in old habits were barriers to change, that mental distress could lead participants to question the efficacy of HLCs [12], and that the feelings of shame and guilt could hinder participants’ taking responsibility for changing their lifestyle behaviours [13]. Taken together, these studies suggest that lifestyle change is a challenging process to initiate and maintain successfully.

Recently, a systematic review underscored the importance of five factors that help promote successful change *and* maintenance of new lifestyle behaviours, namely, having autonomous reasons for the health behaviour change, having skills to monitor and regulate the health behaviour change, making the new lifestyle behaviours habitual, having physical and psychological resources available, and having supportive environments available [14]. It is interesting to note, therefore, that numerous studies using self-determination theory (SDT) have shown that health behaviours that are initiated and regulated with autonomous motivation are more likely to be maintained over time [15, 16]. From the perspective of SDT, the quality of motivation can be distinguished as either more controlled or more autonomous. Controlled motivation involves an experience of pressure or coercion to think, feel, or behave in a particular way, perhaps in order to comply with a health care provider’s request or to avoid shame and guilt for not living in a healthy way [15]. In contrast, autonomous motivation involves an experience of choicefulness, volition, and reflective endorsement of action, such that the individual understands the value of the new lifestyle behaviour and engagement in the behaviour is congruent with a broader set of values and beliefs that the individual endorses. Also, from the perspective of SDT, all individuals require support for satisfaction of the basic psychological needs for autonomy (an experience of self-determination), competence (an experience of effectance), and relatedness (an experience of close connection with others) in order to function in a healthy, integrated way. Importantly, satisfaction of these basic psychological needs is conducive to autonomous motivation, physical health, thriving, and psychological well-being [17, 18].

Other theories and research outside of SDT have elaborated on the process of goal pursuit, and have underscored the importance of planning and executing actions that promote goal attainment as well as resolving cognitive, emotional, and behavioural challenges that impede success [19, 20]. Lazarus [21], for instance, defined coping as the cognitive and behavioural efforts that are used to manage stressful events, and highlighted the importance of emotional coping skills for successful goal pursuit. Ferrer and Mendes [22] underscored the importance of affective states in health decisions, as health-promoting behaviours can be undermined by stress or poor emotion regulation. Altering cognitions to help regulate emotions has been shown to be beneficial in treating obesity [23], and maladaptive cognitions have been shown to be barriers to successful treatment [24]. Behavioural strategies, such as exercise, have been shown to be valuable for emotion regulation, too [25–27]. Lifestyle change is a complex and multifaceted process that requires successful regulation of various experiences and competing motives. Although HLCs might have the potential to promote optimal (or autonomous) motivation for physical activity and diet, and offer guidance on how to plan, execute, and cope with lifestyle changes, there is a gap in knowledge around how the initiation and maintenance of such changes are experienced by participants in HLCs over time. The initiation and maintenance of health behaviour change can be difficult for individuals to disentangle in retrospect, as they tend to experience one continuous process of lifestyle change. Accordingly, the aim of the current study was to examine the factors that participants in an HLC perceive as relevant for the initiation and maintenance of lifestyle changes toward more physical activity and consumption of a healthier diet.

## Method

### Study design and setting

We conducted a qualitative study using individual, semi-structured interviews to examine participants’ experiences with health behaviour change after participating in an HLC to increase physical activity and improve quality of diet. This approach afforded an opportunity to explore the participants’ own experiences and perceptions [28] in order to create a broad, deep understanding of the factors that are relevant for initiation and maintenance of lifestyle changes toward more physical activity and consumption of a healthier diet. The interviews were conducted at the end of a multi-method research project that lasted for 12 months in one HLC. The aim of this project was to examine perceptions of need support from the HLC as well as changes in autonomous motivation and perceived competence (constructs relevant to SDT); changes in physical activity and diet (health behaviours); and changes in visceral fat, lower body strength, and psychological distress (physical and mental health outcomes). Inclusion criteria for this project included being a Norwegian-speaking citizen who is at least 18 years old and consulting an HLC in order to improve physical activity and diet. Participants were excluded due to severe and disabling mental illness.

Individuals were able to attend the HLC either through self-referral or through referral by a general practitioner, health worker, or social worker. Individuals who attended the HLC were given choice of activities (to support autonomy) from a menu of options that enhance perceived competence for lifestyle change (to support competence), including lifestyle courses (weekly for 14 weeks), walking groups (continuously ongoing), food classes (weekly for 5 weeks), yoga (eight sessions), training groups (continuously ongoing), and discussion groups (five sessions). Also, individual face-to-face guidance was offered on goal setting and goal striving. All activities and guidance were provided by a team that was comprised of physiotherapists, dietitians, a social worker, a nurse, a peer, and a sports educator. The HLC employees were experienced in lifestyle counselling and trained to foster good team spirit in groups (to support relatedness). Participants were given free choice in selecting from the menu of options and could participate in the continuously ongoing groups as desired. In addition, participants were encouraged to engage in physical activities on their own.

### Participants

Of the 120 individuals who were recruited for the 12-month research project, 50 participants completed the 12-month follow-up assessment. Potential participants were asked by their counsellors if they were willing to complete an individual, semi-structured interview, and recruitment ended with the completion of 14 interviews due to sufficient information power [29]. The counsellors did not record participants’ reasons for declining to take part in the interview, and several participants had yet to complete the 12-month follow-up assessment even after 14 interviews had been completed and information power was considered to be sufficient, which means that recruitment for the current study could have continued if data saturation was weak. Potential participants were recruited into the current study regardless of how successful they were with their lifestyle change. Table 1 presents sample characteristics.

**Table 1:** Sample characteristics

Gender	
Male	6
Female	8
Age (in years)	
20–30	4
30–40	4
40–50	2
50–61	4
Education	
Completed secondary school	8
Completed bachelor’s degree or higher	6
Diagnosis (self-reported)	
Yes (fibromyalgia, ileostomy, diabetes, high blood pressure, asthma, chronic kidney impairment, vocal cord impairment, narcolepsy, depression, ADHD, and/or dystonia)	8
No	6
Work status	
100% capacity	6
Reduced capacity	4
Student	2
Sick leave	2
BMI^a^ category (value)	
Normal weight (18.5–25)	2
Overweight (25–30)	1
Obese (> 30)	11
Symptoms of mental distress^b^	
Yes	5
No	9

^a^ BMI category [30] was measured by Inbody 720 (Inbody 720, Body Composition Analyzer, Biospace Co. Ltd.)

^b^ Measured by the Hopkins Symptom Checklist [31]

### Ethics

The Regional Committee for Medical and Health Research Ethics in Norway concluded that ethics approval was not necessary for this study, and the Data Protection Officer in the Municipality of Stavanger and the Norwegian Data Protection Authority granted permission for collecting and storing the data. All participants provided informed written consent after receiving information about the purpose and procedures of this study as well as how data would be handled in a secure way. All data were permitted to be stored for 10 years in a manner that is secure and protects privacy.

### Data collection

The individual, semi-structured interviews were conducted “on site” by the first author, who had previous experience working in the field at the HLC. On average, interviews took 42 min to complete (range: 21 min to 100 min). Throughout each conversation, the interviewer aimed to be open, respectful, and non-judgemental in order to create an atmosphere of trust that would ensure that the participant felt cared for, and to enhance the quality of the interview [32]. Each interview followed a guide that was developed for the current study and designed to be open to various experiences and not based on theoretical assumptions. It was comprised of five parts. 1) “Introduction”, in which the purpose of the study was described, and anonymity was assured. 2) “Background”, in which participants were invited to discuss their motives, goals, and previous experiences around lifestyle change. 3) “Experiences from the Last Year”, in which participants were asked to discuss their experiences with physical activity and diet during the last year, including any change attempts and strategies for change, physical and mental health correlates, group and/or individual counselling, perceptions of competence and support, goal development and pursuit, and methods of assessment. 4) “Reflection Themes”, in which participants were invited to reflect on their perceptions of health, motivation, identity, and movement in and out of periods of managing lifestyle changes or not. 5) “Closing”, in which participants were asked to imagine that they had 2 min to share their experiences with lifestyle change with a beginner, and to do so aloud. Audio recordings of the interviews were transcribed verbatim and then checked for accuracy.

### Data analysis

After each interview, the first author listened to the audio recording and reflected upon the themes contained therein. The co-authors contributed to the process of data analysis as follows: one read all of the material, two read half of the material, and two read large amounts of quotes. The co-authors contributed to the search for themes, to the sorting of data into themes and subthemes, to coding, and to linking themes with theory, if applicable. All co-authors agreed on the final version of the data analysis. The co-authors had unique backgrounds and perspectives relevant to themes and subthemes. Data analysis was performed using Systematic Text Condensation (STC) described by Malterud in 2012 [33], which is a systematic and valid method for the analysis of qualitative data. STC was used to analyse the meaning and content of data across the set of participants, which gave the possibility for abstraction beyond the interview narrative. Although the interviews were conducted at one time point and thus have limited capacity for exploring processes over time, this limitation was mitigated by the longitudinal design of the multi-method research project and participants’ retrospective recollections [28, 33].

The analysis of qualitative data using STC proceeds through four phases. In Phase 1, the goal was to obtain an overview of the data and define preliminary themes—to “search for patterns in the chaos”, which included searching for patterns in the experiences based on demographic characteristics. In Phase 2, the preliminary themes were developed further into subthemes/codes by sorting meaning units (i.e., text fragments relevant to the research question) drawn from the preliminary themes. In Phase 3, the decontextualized meaning units were coded into subgroups regardless of the individual participants from whom the meaning units were derived. In Phase 4, main themes were synthesized to ensure that the results represented the original context in such a way that protected the validity and wholeness of the original context. Also in Phase 4, an analytic text was written that described the “story” told by the material and illuminated the text with meaning units [28, 33].

## Results

During the individual, semi-structured interviews, participants reflected on their experiences around striving to increase their physical activity and consume a healthier diet in consultation with the HLC, as well as the cognitive, behavioural, and emotional skills that they perceived to be important for the initiation and maintenance of these lifestyle changes. The conclusions on the findings were made regardless of the participants’ demographic categories and resulted in three main themes, namely, 1) motivational basis for change, 2) coping for emotional balance, and 3) self-regulation skills. Table 2 presents examples of meaning units for the main themes and subthemes. In general, the participants considered a healthy lifestyle to be an important foundation for better health, and their progress toward lifestyle change tended to co-occur with an enhanced sense of well-being and thriving. Yet their pursuit of a healthy lifestyle was also described as an ongoing process with emerging challenges along the way. The presentation of results is supplemented with quotes from participants along with their interview number, gender, and age category.

**Table 2:** Examples of meaning units for the main themes and subthemes

Main Themes	Subthemes	Meaning Units [Valence]
**Motivational basis for change**	**Controlled versus autonomous motives for healthy behaviour**	*Of course, I had thoughts that I should go for walks and eat healthy. But it hasn’t been, like, for real. Not an urge, nor an extra feeling that I should do it. It has just been a standard, yes, but you should do this and should really … (5,M,20–25)* [Negative]
		*It was not me who made contact directly, it was my father. He is a doctor … he thought it was for me, and he signed me in … It was a surprise to me … I wanted better health … one looks better with better health … If I could get hypnotized, and just fixed in a snap, it would be brilliant. (4,M,30–35)* [Negative]
		*The reason all this started is that I realized that much of this is about my health. And I am not going to the Olympics … Health is for me to feel that the everyday tasks are OK, with no fuss of being too heavy … So, health is that I can function … (13,F,45–50)* [Positive]
		*I just want to live, to have more energy in a way. More, feel that I am a healthy, strong man. (9,M,35–40)* [Positive]
	**Relational support**	*That course was a gift from above … because there are so many pitfalls to tremble in … so it has been amazing … and I have learned about exercise and we had an exercise session after class that was fantastic. (14,M,35–40)* [Positive]
		*It was good to come here, something to go to every week, having the focus … Now I have stopped everything … maybe due to not coming here (the HLC), not committing …*. *Not many offerings for fat and slow people. (3,M,50–55)* [Negative]
**Coping for emotional balance**	**Cognitive appraisals**	*I drank my first glass of water in a very long time. It has mostly been Coke. Previously I have thought “yucky” - it tastes like nothing … But this time when I drank the water, I imagined the liver and kidneys cheering and enjoying themselves. Finally, something clean … It was not as good as Coke of course, the first time. But at least someone was happy. (14,M,35–40)* [Positive]
		*But there is a kind of perfectionism in it, which implies that if it is not perfect – no, then we must throw it all away. Zero or nothing almost. I wish I could control it and manage it. Because if I could then I would know that 95 is just as good as 100, everything is better than 0, even if it was 30 or 20 or 1, it is better than 0. But I cannot manage because when it starts to crumble, below 100, closer to 90, closer to 80 – my motivation withers and it falls apart. (5,M,20–25)* [Negative]
		*All this of being fat and overweight is kind of shameful because it is a societal focus on the body … Why don’t you pull yourself together and do something about it? Like, you’ll die of it. So it is very coated with shame because it involves your identity. Like who are you? Okay, when you let it slip there, how are you in other areas? Are you stupid? … You are almost expected to run a marathon before anyone bothers to talk to you … (3,M,50–55)* [Negative]
	**Behavioural adjustments**	*I have had an eating disorder … but I have it mostly under control now … when you are full you are full – you don’t have to eat until you throw up … Still, if I have a bad day or a painful emotion then I want to go home and eat chocolate pudding … now I go for a walk … take a shower … eat vanilla protein pudding … Find other ways to cope. (6,F,20–25)* [Positive]
**Self-regulation skills**	**Goal setting**	*I had a goal to reduce my weight … Then I tried to think about waist circumference because that was smart. But the conclusion was that, in the end, I was not going to have any goals regarding kilograms at all, I have to focus on lifestyle … if I focus on that and manage to adhere for the long-term then I will decrease weight anyway. (9,M,35–40)* [Neutral]
	**Creating space for new behaviours**	*I care a lot for people, for good and bad, right. I do not think it has changed, I have just become more aware of, even if you are a kind person you do not have to put yourself last. I used to do that. (6,F,20–25)* [Positive]
		*It is rolling back to my old life … I am not getting it done … I must look after my job … Life gets in the way, so to speak. (3,M,50–55)* [Negative]
		*It is an epiphany when you realize that your children understand that you must (spend time exercising), you need this. Like, you don’t have to feel bad. 1,F,55-60* [Positive]
	**Monitoring satisfaction with outcomes**	*It would be great if one could get motivation from others, but basically you must be aware of getting motivated yourself. You get that if you work out and experience the joy it gives, then you feel more vigilant, happier. When you eat healthy you feel more vigilant. (1,F,55–60)* [Positive]
		*The tests were good, I liked them … It was useful to get these, literally, what you need. And maybe look fat percent related to what you managed of the exercise stuff … push ups and things I could do. (8,F,60–65)* [Positive]
		*In those assessments, you get confronted with a shameful side of yourself. Which is kind of good because it forces you to, it gets clearer than when pushed aside … (3,M,50–55)* [Neutral]

### Main theme 1: motivational basis for change

The first main theme focused on the motives behind initiation and maintenance of lifestyle changes along with the importance of a relationally supportive environment to promote perceived competence in pursuing a healthy lifestyle. This main theme encompasses two subthemes, namely, 1) controlled versus autonomous motives for healthy behaviour and 2) relational support.

#### Subtheme 1.1: controlled versus autonomous motives for healthy behaviour

Some participants made a health behaviour change in response to external pressure, such as a spouse, family member, friend, doctor, childhood recollection, or societal/media standard, which tended to be perceived as controlling. Some of these participants felt able to maintain their healthy lifestyle over time, yet the motivation for their lifestyle changes was not autonomous. As a result, maintenance of the new behaviours became difficult after the external pressure was removed.

*But, my up-going spiral, in the period I stayed with my grandma, it was not really me pushing it upwards. I just “turned off” and did what I was told … so in retrospect, when I moved to live by myself it is natural that the spiral started to go down because I was not the one pushing it up … I cannot make that a standard since I got all the help to get the spiral moving up … I must learn myself how to do it. 5, M, 20-25*

Other participants made a health behaviour change by doing things that they did not like, or by engaging in lifestyle behaviours that held no personal value or were experienced as unpleasant.

*I have never enjoyed exercise. I think it is horrible … I never find it lovely or fun. 3, M, 50-55*

Reflecting more autonomous reasons for healthy behaviour, most participants identified their health as an important motive behind the initiation of lifestyle changes. Indeed, the process of health behaviour change was fuelled by motives that were personally important and valuable for living a good life, rather than to achieve an appealing image. Participants felt satisfied as they developed a sense of congruence between their perceived identity and lifestyle commitments, which aided their maintenance of a healthy lifestyle.

*I have spent a lot of time reflecting on whether I am a person who exercises or not … Maybe I have become a person who exercises? I believe I have, because I really enjoy it, I do it because I enjoy it. It makes me feel good, and I do it because it is fun doing it. 13, F, 45-50*

This congruence between perceived identity and lifestyle commitments afforded participants a sense of confidence to set their own standards for success, rather than using external standards for their evaluation of success.

*It is more important for me to be a person who stands up for myself, who is confident about my feelings, than to lose weight. (10, F, 30–35)*

Indeed, such congruence promoted the emergence of a “new” identity that was fulfilling, satisfying, and vitalizing, and this energy could be used for the benefit of others and to promote the maintenance of lifestyle changes.

*Slowly I am turning into a “Mr. Healthy” who has the energy to vacuum when I get home after a long day, who goes for walks and helps when needed … I am not just the “Fatty”—a little “Mr. Healthy” too. (14, M, 35–40)*

#### Subtheme 1.2: relational support

Many participants found it necessary and valuable to seek out and utilize relational support from others, even though some struggled to find sustainable sources of such support. Participants identified their family, friends, work colleagues, group members at the HLC, and employees of the HLC as sources of relational support. Indeed, guidance from HLC staff was perceived as professional, knowledgeable, affirming, and effective at revealing internal struggles and increasing competence.

*[The individual counselling] was very good … it was the first time I spoke to someone about my problems regarding weight and exercise. Ever. So, it was challenging because I found it hard to open up … I joined a class on emotions and eating when I realized that it was about an eating disorder, not just laziness or lack of knowledge. (10, F, 30–35)*

Also, participants experienced their relationships with group members at the HLC to be helpful for raising awareness, staying focused, and feeling connected.

*I joined a structure group … here the focus is on what you do and what you don’t do ... And the focus gets so big, it forces you to think about changes that work. So, the structure group helped me most. (9, M, 35–40)*

### Main theme 2: coping for emotional balance

The second main theme focused on strategies for coping with the challenges and potential pitfalls to lifestyle change that were associated with stress, poor self-image, sickness, injuries, problematic relationships, loneliness, depression, and negative life events. Behaviour change was perceived as a complex process that is affected by time, context, physical illness, and mood. It was, thus, necessary for participants to use coping strategies to achieve emotional balance. This main theme encompasses two subthemes, namely, 1) cognitive appraisals and 2) behavioural adjustments.

#### Subtheme 2.1: cognitive appraisals

Several participants described how they became aware of and changed their cognitive patterns in order to strengthen their health behaviour change, while others felt stuck in “old ways” of thinking. Often, it was necessary to acquire new cognitive techniques such as positive visualization, reducing catastrophic thinking, replacing self-blame with self-compassion, and altering a black-and-white (or dichotomous) thinking style.

*I am in a totally different path than a year ago. I had a black-and-white mind set; if you eat chocolate one day, three weeks are ruined … I got a lot of help because I have struggled with the black-and-white mind set. (6, F, 20–25)*

It was a common experience for participants to struggle with high expectations, and some participants dealt with this struggle by lowering their expectations in order to make their goals more attainable.

*I have learned I just have to schedule. I am going to exercise on Wednesday, I am going to exercise on Sunday. And I have learned to go on no matter what. Like, you don’t have to examine, am I in too much pain to go? Am I too tired to go? You go. Then you can stop if you cannot manage and go home. But always go. Try. And most times it is OK. Usually you manage to complete that exercise. (1, F, 55–60)*

Some participants used techniques such as mindful eating and focusing on food added rather than food removed in order to deal with experiences of deprivation due to changes in diet.

*If you start changing by denying yourself things, then you will crack at the end. Try instead to****add****things instead of removing … I can eat carrots, I can eat broccoli, I can eat fish and salmon. And it has helped a lot … (10, F, 30–35)*

Many participants experienced shame—an unpleasant feeling of not “meeting the standard” of others and/or society. Indeed, it was deeply satisfying for participants to liberate themselves from the experience of shame, which promoted their maintenance of a healthy lifestyle.

*And I had a lot of barriers. I could not go to the swimming pool, I could not get undressed in front of others, I was afraid to sit on chairs … But the shower was filled with women of all sizes … And it was so nice, they were really all sizes and I found it fantastic … they were very relaxed. So, I felt relaxed at once when I got in, I did not mind, and went out to the pool … It all went so much better than I imagined, and it occurred to me that I might be rid of that enormous panic for those things. That I have won over it a long time ago, I just did not know, did not believe it. (13, F, 45–50)*

#### Subtheme 2.2: behavioural adjustments

For some participants, the desire to escape from stressful life circumstances that were marked by multiple challenges, relational troubles, and feelings of depression and anxiety was overwhelming, which made it difficult to focus on lifestyle changes. Unfortunately, some participants tried to mute the intensity of these painful experiences with alcohol, tobacco, emotional eating, and/or gambling.

*It is my “escape”, kind of … Gambling and food. Overeating … It is what I use to avoid thoughts … (5, M, 20–25)*

Still, many participants who struggled with such pain made behavioural adjustments that were in line with more sustainable ways to cope, including seeking comfort in nature, socializing, reading, yoga, meditation, physical activities, and eating healthy snacks. Indeed, these alternative strategies were discovered by the participants themselves and/or through guidance from HLC personnel and other health services.

*I still have the yoga. It does a lot for wellness. For the mind. Actually, I think it is the yoga that has given me most throughout the year. It is like there is a little gap to the intense feelings attached to food. Which makes me able to analyse it, if not in the moment at least in retrospect. (10, F, 30–35)*

*I find [exercise] joyful and the effects are good, both physically but not at least mentally … regarding depression. (11, M, 25–30)*

### Main theme 3: self-regulation skills

The third main theme focused on several specific skills that were helpful to the initiation and maintenance of lifestyle changes. This main theme encompasses three subthemes, namely, 1) goal setting, 2) creating space for new behaviours, and 3) monitoring satisfaction with outcomes.

#### Subtheme 3.1: goal setting

Although better health was an overarching goal, most participants also decided on and developed specific short-term goals related to lifestyle changes, rather than (for example) weight, during their time at the HLC. Indeed, many participants viewed weight as an outcome of health behaviour change.

*I had eating supper as a main goal. And increased physical activity. Those two. I know if I can manage them, I will manage to change my lifestyle in the long run anyhow. 9, M, 35-40*

Still, initially some participants viewed health behaviour change as having instrumental value for the overall goal of weight control, such that the reason to exercise and eat healthy was to control weight. Yet over the year of participation at the HLC lifestyle changes tended to be viewed as having inherent value.

#### Subtheme 3.2: creating space for new behaviours

In order to pursue their lifestyle changes, participants found it important to create space for new behaviours, which are time consuming and could affect relationships. For example, time spent exercising leaves less time for family, and acceptance of this fact was worthwhile.

*It is an epiphany when you realize that your children understand that you must [spend time exercising], you need this. Like, you don’t have to feel bad. (1, F, 55–60)*

*Spend more time planning what to buy, and make sure to only have those healthy options … I spend time doing groceries, I spend time cooking, and I experience it does not take too much time. And the results are so good, the body feels better. (8, F, 60–65)*

Without creation of this “space”, the lifestyle changes were vulnerable to being abandoned in the wake of daily activities and poor planning.

*I see myself as the hindrance … Because I am really good at not implementing my plans, at least if I have made bad plans. (4, M, 30–35)*

#### Subtheme 3.3: monitoring satisfaction with outcomes

Many participants expressed vigilance as an awareness of their routines and evaluation of their outcomes in order to assist with their progress.

*Awareness of what I am doing has been very important for me. (7, F, 20–25)*

Participants tended to experience joy and a sense of competence as they progressed toward the initiation and maintenance of lifestyle changes.

*Exercise has given me a sense of mastery that has affected other areas … It is both that it works out OK and that I am not as paralyzed because I don’t think it will work out. (11, M, 25–30)*

Participants experienced the assessments that occurred at the HLC (body composition and health related fitness) in a variety of ways. Many participants viewed these assessments as concrete measures of progress—or lack thereof—that was important information for goal striving. A few participants felt shame in light of these assessments, too.

*I found the machine [that measured body composition] super. It measured progress when I did not measure it myself ... It was reassuring that what I do is not completely meaningless. (4, M, 30–35)*

For some participants, their satisfaction with outcomes was a function of psychological wellness rather than weight-related changes.

*No, I think it has turned out fine. I know that the results regarding the research havn't turned out that good. But if I were to be tested again in a year it would show quite big changes. (9, M, 35–40)*

## Discussion

The aim of the current study was to examine the factors that participants in an HLC perceived to be relevant for the initiation and maintenance of lifestyle changes. The main findings underscored the importance of 1) a motivational basis for change, 2) coping for emotional balance, and 3) self-regulation skills for the successful pursuit of a healthy lifestyle. Below, we reflect on each of these main themes.

### Motivational basis for change

Most participants in our study expressed a heightened sense of being “in charge” of their health behaviour change such that the motives for the lifestyle changes emanated from the self rather than from a source external to the self. Although some participants initiated their health behaviour change due to someone else’s desire or demand, over time such behaviours tended to be given up or their motives were internalized and experienced as more autonomous. This is particularly important because autonomous motivation for health behaviour change was not only satisfying but conducive to maintenance of lifestyle changes as well. Indeed, this finding is consistent with SDT [15, 34]. Participants found it to be depleting and difficult to maintain health behaviour change when their motives for lifestyle changes were strongly linked to societal standards and/or social pressures. With such controlled motivation, participants tended to feel like a “puppet on a string”, which is unlikely to promote maintenance of lifestyle changes over time [15, 34].

Specifically, with regard to consumption of a healthier diet, some research outside of SDT has revealed a comparable set of findings. Sarlio-Lahteenkorva [35], for instance, found that dietary self-regulation was difficult without adequate structured support, and Hindle and Carpenter [36] found that autonomous motivation for a healthy diet tended to co-occur with the maintenance of a healthy diet. As the desire to eat healthy was common for all participants in our study regardless of weight category, SDT can offer a broad perspective for understanding the motives behind healthy eating and disordered eating behaviours [37].

Likewise with regard to physical activity, the health-enhancing, stimulating, and pleasurable facets of exercise have been shown to promote maintenance of an active lifestyle [26, 27]. Some of the participants in our study experienced a transition toward a new identity as “an exerciser” that aided their maintenance of a healthy lifestyle. Interestingly, Eynon and colleagues [38] found identity transformation to be conducive to maintenance of lifestyle changes among participants who received an exercise referral program, while others have reflected on the potential of identity elaboration to promote health [39]. Directly in line with our findings, Texeira and colleagues [40] reported that a motivational profile marked by a high level of autonomous motivation was helpful for maintenance of physical activity. Thus, it is important for health professionals to be aware of autonomous (versus controlled) motives in those with whom they work and promote competence to explore autonomous reasons for lifestyle changes [40], as such internalization can provide a solid foundation for sustained health behaviour change over time.

Most participants in our study expressed the importance of the social environment as a source of relational support, which they obtained from their family, friends, or work colleagues. Also, group members and employees at the HLC were perceived as supportive. Participants described the HLC as important for their health behaviour change because it supported their competence for lifestyle changes. SDT underscores the importance of relatedness, as individuals are more likely to adopt values and behaviours that are promoted by those they trust and to whom they feel connected [15]. It is unfortunate, therefore, that shame can hinder individuals’ seeking relational support, such as the participant who did not find groups outside of the HLC for the “fat and slow”, as it was expressed. Sagsveen and colleagues [41] found that relational continuity was important to individuals who utilize HLC services, which can be an experience that is challenging to guarantee in an ever-changing clinical practice. Nonetheless, it is important that clinical systems are developed to support the basic psychological needs of those who utilize HLC services. For instance, although some might prefer that HLC providers are knowledgeable “experts” [41], it is critical that the HLC providers are flexible in their guidance around the implementation of changes in participants’ lives. In line with SDT [42], we recommend that HLC providers work in partnership with their participants to inform and support autonomous decision making.

### Coping for emotional balance

Several participants in our study expressed the value of altering cognitive appraisals, especially when the lifestyle changes were experienced as challenging, depleting, or overwhelming. Lazarus [21] defined the term cognitive appraisal as one’s personal interpretation of and response to a stressful situation. Cognitive appraisals are evaluations of possibilities when faced with challenge or threat that can lay a cognitive foundation for coping with stressful events as they occur. Indeed, the appraisal that an individual makes of a stressful situation can affect the response to relapse in the context of health behaviour change—either to accept the relapse and continue moving toward a healthy lifestyle, or to give up in the face of perceived failure. Of note, cognitive appraisals can be conscious or non-conscious—intuitive and automatic. Interestingly, some participants experienced satisfaction as they uncovered and re-evaluated their cognitive patterns, as doing so provided tools for managing their health behaviour change and maintenance. This is encouraging, as if HLCs can promote internal strength in participants through the alteration of their cognitive appraisals then the possibilities for maintenance of lifestyle changes might increase. Similarly, research has shown that adding cognitive therapy to standard obesity treatment is beneficial for psychological health, behavioural persistence, and weight control over time [23]. If ignored, then maladaptive cognitions can function as “tripwires” as shown in a large qualitative study by Byrne and colleagues [24], who found that overweight individuals who struggled to maintain a healthy lifestyle were “stuck” in a dichotomous thinking style.

Most participants in our study expressed that they made behavioural adjustments (in addition to altering cognitive appraisals) as a strategy for coping with painful experiences—from muting the painful experiences with, for example, overeating to more healthy strategies described below. Byrne and colleagues [24] found that relapses among overweight individuals who struggled to maintain a healthy lifestyle were associated with eating behaviour that was used to regulate mood and manage stressful life events, and some participants in our study described being “stuck” in such patterns. One emotion that was especially difficult for participants to manage was shame, as fear of being body shamed and objectified for being overweight could trigger avoidance of health-promoting activities and situations [43]. Indeed, often shame is a central part of how overweight and obese individuals conceptualize themselves, as they tend to feel guilty for having the “wrong” lifestyle [13]. Although difficult, some participants expressed the possibility for liberation from the perceived condemnation from others, which enhanced their competence for and commitment to lifestyle changes. Ferrer and Mendes [22] described emotion regulation and coping processes as more effective than risky behaviours such as binge eating and drug use to downregulate unpleasant affect. Finally, some participants made behavioural adjustments such as hiking in the peace of nature, socializing, yoga, and physical activities, which is congruent with research showing that exercise can upregulate mood [25] and that positive affect can enhance autonomous motivation [15, 34, 44].

### Self-regulation skills

Most participants in our study expressed the importance of goal setting, creating space for new behaviours, and monitoring satisfaction with outcomes as key factors that were relevant for the maintenance of health behaviour change. Although participants viewed health as an overarching goal, they also had specific short-term goals to assist in their process toward lifestyle changes, including to eat supper every day and go for a 30-min walk every day. Successful goal setting involves deciding the goals to pursue and the criteria for success or failure [19], which has been shown to be effective for behaviour change [45], although previous research on goal setting has been equivocal [46]. Interestingly, SDT makes an important distinction between goals that have intrinsic content and are conducive to basic psychological need satisfaction and well-being versus goals that have extrinsic content and can frustrate basic psychological needs and yield ill-being [18, 42]. With this distinction, it might be that setting extrinsic goals is unassociated or inversely associated with goal attainment, whereas setting intrinsic goals is conducive to health behaviour change and maintenance [47].

Further, participants in our study expressed the importance of thorough planning and making prioritizations for goal pursuit. Lifestyle changes can be time consuming and can affect relations with others, and indeed conflicting identities (for example, parent versus exerciser) can undermine the behaviours on which a healthy lifestyle is predicated even when disease risk is present [48]. Therefore, thorough planning and prioritization can “bridge the gap” between cognition and action, encourage the individual to think prospectively about the future, and reveal obstacles and challenges that might otherwise impede success at attaining goals [20].

Most participants in our study expressed the value of monitoring and evaluating their goal progress, as satisfaction, or lack thereof, with outcomes fueled ongoing goal pursuit and prompted the necessary adjustments to promote success. This finding is aligned with the work of Rothman [49], who suggested that the initiation of new behaviours is based on *expectations* of success whereas the maintenance of new behaviours is based on *satisfaction* with one’s achievements. Also, in line with our findings, Rothman suggested that high expectations at the outset can create problems for continued pursuit of new behaviours, as one might be dissatisfied with the outcomes if the standards are set too high. Indeed, Byrne and colleagues [24] found that dissatisfaction with weight-related outcomes can increase the likelihood of relapse among overweight individuals, such that too-high expectations can hinder the maintenance of healthy lifestyle behaviours.

### Strengths and limitations of the study

The findings from the current study provide subjective, first-person accounts that extend knowledge gained from the quantitative literature on the initiation and maintenance of lifestyle changes. Although the sample was of medium size, as typical sample sizes for such research tend to vary from 5 to 25 participants [32], the information power can be regarded as rich because the aim was narrow, the participants held characteristics that were truly specific to the aim, the study was grounded in an evidence-based theoretical perspective (namely, SDT), and the dialogue from the qualitative interviews was of high quality [29]. Still, the transferability of the findings would be stronger if the sample size was larger and the informants came from various HLCs. Nevertheless, the variation in age, gender, employment status, weight category, and mental health in our sample reflected the diversity found in other HLCs, which enhances the representativeness of the findings [9, 50, 51]. That being said, it is important to note the large amount of dropout from the multi-method research project as a limitation, such that participants who were in the current study could have held different perspectives than those who left the project. Still, the current study makes an important contribution even with this limitation in mind.

Investigators with different backgrounds were involved in data analysis, which encouraged the emergence of diverse perspectives prior to the conclusion on themes. The inputs that were made at different phases of the analysis process have been saved and are available for review in order to enhance the credibility of the findings. One important limitation, though, is that there was no follow-up with the participants. Also, the current study did not distinguish the processes of initiation and maintenance of health behaviour change in participants’ experiences.

The broad use of quotes in the Results section affords the reader an opportunity to confirm—or disconfirm—the findings [51]. The interviewer was forthcoming about her work in an HLC, but this could have left some participants with the desire to “decorate” their experiences. That being said, it is important to highlight the fact that participants offered rich accounts of their experiences with success *and* failure in an HLC.

## Conclusion

The current study enhanced an understanding of the initiation *and* maintenance of lifestyle changes, although these processes were not disentangled in participants’ experiences. In line with SDT, the results suggested that lifestyle change is more likely to be initiated and maintained when goals are not only achievable but also regulated with autonomous motivation and of intrinsic value. Conversely, lifestyle change is difficult to maintain when motives are external to the self. Further, cognitive and behavioural skills were valuable and necessary in coping with unpleasant emotions. Finally, the critical function of self-regulation skills for making realistic plans and prioritazations in order to balance healthy lifestyle behaviours with the routines of “daily life” while monitoring outcomes was readily apparent. Healthy Life Centres can contribute to these processes in meaningful ways.

## Data Availability

The data used in the current study are not publicly available, yet they can be made available (anonymized) by CHS upon reasonable request so long as permission for data storage is applicable.

## Abbreviations

NCD: Non-Communicable Disease
HLC: Healthy Life Centre
SDT: Self-Determination Theory
BMI: Body Mass Index
STC: Systematic Text Condensation

## References

[1] GBD 2015 Risk Factors Collaborators (2016). Global, regional, and national comparative risk assessment of 79 behavioural, environmental and occupational, and metabolic risks or clusters of risks, 1990–2015: A systematic analysis for the Global Burden of Disease Study 2015. Lancet.

[2] World Health Organization (2013). Global action plan for the prevention and control of noncommunicable diseases 2013–2020.

[3] Loyen A, Clarke-Cornwell AM, Anderssen SA, Hagströmer M, Sardinha LB, Sundquist K (2017). Sedentary time and physical activity surveillance through accelerometer pooling in four European countries. Sports Med.

[4] Guthold R, Stevens GA, Riley LM, Bull FC (2018). Worldwide trends in insufficient physical activity from 2001 to 2016: a pooled analysis of 358 population-based surveys with 1.9 million participants. Lancet Glob Health.

[5] World Health Organization. Global Health Observatory (GHO) data: Unhealthy diet. 2020. Retrieved from https://www.who.int/gho/ncd/risk_factors/unhealthy_diet_text/en/.

[6] Fjeldsoe B, Neuhaus M, Winkler E, Eakin E (2011). Systematic review of maintenance of behavior change following physical activity and dietary interventions. Health Psychol.

[7] Denison E, Underland V, Berg RC, Vist GE (2014). Effects of more than three months organized follow-up on physical activity and diet for people with increased risk of lifestyle related disease. Report from Norwegian Knowledge Centre for the Health Services (NOKC) No. 16.

[8] The Norwegian Directorate of Health (2016). Veileder for kommunale frisklivssentraler. Etablering, organisering og tilbud. [Recommendations for establishing, organizing and content of municipal healthy life centres.].

[9] Samdal GB, Meland E, Eide GE, Berntsen S, Abildsnes E, Stea TH (2019). The Norwegian healthy life Centre study: a pragmatic RCT of physical activity in primary care. Scand J Public Health.

[10] Lerdal A, Celius EH, Pedersen G (2013). Prescribed exercise: a prospective study of health-related quality of life and physical fitness among participants in an officially sponsored municipal physical training program. J Phys Act Health.

[11] Følling IS, Kulseng B, Midthjell K, Rangul V, Helvik A-S (2017). Individuals at high risk for type 2 diabetes invited to a lifestyle program: characteristics of participants versus non-participants (the HUNT study) and 24-month follow-up of participants (the VEND-RISK study). BMJ Open Diabetes Res Care.

[12] Følling IS, Solbjør M, Helvik A-S (2015). Previous experiences and emotional baggage as barriers to lifestyle change - a qualitative study of Norwegian healthy life Centre participants. BMC Fam Pract.

[13] Salemonsen E, Hansen BS, Førland G, Holm AL (2018). Healthy life Centre participants’ perceptions of living with overweight or obesity and seeking help for a perceived “wrong” lifestyle - a qualitative interview study. BMC Obesity.

[14] Kwasnicka D, Dombrowski SU, White M, Sniehotta F (2016). Theoretical explanations for maintenance of behaviour change: a systematic review of behaviour theories. Health Psychol Rev.

[15] Ryan RM, Patrick H, Deci EL, Williams GC (2008). Facilitating health behaviour change and its maintenance: interventions based on self-determination theory. Eur Health Psychol.

[16] Teixeira PJ, Carraça EV, Marques MM, Rutter H, Oppert J-M, De Bourdeaudhuij I (2015). Successful behavior change in obesity interventions in adults: a systematic review of self-regulation mediators. BMC Med.

[17] Deci EL, Ryan RM, Ryan RM (2012). Motivation, personality, and development within embedded social contexts: an overview of self-determination theory. The Oxford handbook of human motivation.

[18] Deci EL, Ryan RM (2000). The "what" and "why" of goal pursuits: human needs and the self-determination of behavior. Psychol Inq.

[19] Mann T, de Ridder D, Fujita K (2013). Self-regulation of health behavior: social psychological approaches to goal setting and goal striving. Health Psychol.

[20] Maes S, Karoly P (2005). Self-regulation assessment and intervention in physical health and illness: a review. Appl Psychol.

[21] Lazarus RS (2006). Stress and emotion: a new synthesis.

[22] Ferrer RA, Mendes WB (2018). Emotion, health decision making, and health behaviour. Psychol Health.

[23] Werrij MQ, Jansen A, Mulkens S, Elgersma HJ, Ament AJ, Hospers HJ (2009). Adding cognitive therapy to dietetic treatment is associated with less relapse in obesity. J Psychosom Res.

[24] Byrne S, Cooper Z, Fairburn C (2003). Weight maintenance and relapse in obesity: a qualitative study. Int J Obes Relat Metab Disord.

[25] Emerson JA, Dunsiger S, Williams DM (2018). Reciprocal within-day associations between incidental affect and exercise: an EMA study. Psychol Health.

[26] Andersen P, Lendahls L, Holmberg S, Nilsen P (2019). Patients’ experiences of physical activity on prescription with access to counsellors in routine care: a qualitative study in Sweden. BMC Public Health.

[27] Bombak AE (2015). Obese persons’ physical activity experiences and motivations across weight changes: a qualitative exploratory study. BMC Public Health.

[28] Malterud K (2011). Kvalitative metoder i medisinsk forskning: En innføring. 3. utgave ed.

[29] Malterud K, Siersma VD, Guassora AD (2016). Sample size in qualitative interview studies: guided by information power. Qual Health Res.

[30] Seidell JC, Flegal KM (1997). Assessing obesity: classification and epidemiology. Br Med Bull.

[31] Strand BH, Dalgard OS, Tambs K, Rognerud M (2003). Measuring the mental health status of the Norwegian population: a comparison of the instruments SCL-25, SCL-10, SCL-5 and MHI-5 (SF-36). Nordic J Pschychiatry.

[32] Kvale S, Brinkmann S (2009). Det kvalitative forskningsintervju. 2. utgave ed.

[33] Malterud K (2012). Systematic text condensation: a strategy for qualitative analysis. Scand J Public Health..

[34] Ng JYY, Ntoumanis N, Thøgersen-Ntoumani C, Deci EL, Ryan RM, Duda JL (2012). Self-determination theory applied to health contexts: a meta-analysis. Perspect Psychol Sci.

[35] Sarlio-Lahteenkorva S (1998). Relapse stories in obesity. Eur J Pub Health.

[36] Hindle L, Carpenter C (2011). An exploration of the experiences and perceptions of people who have maintained weight loss. J Hum Nutr Diet.

[37] Verstuyf J, Patrick H, Vansteenkiste M, Teixeira PJ (2012). Motivational dynamics of eating regulation: a self-determination theory perspective. Int J Behav Nutr Phys Act.

[38] Eynon MJ, O’Donnell C, Williams L (2018). Gaining qualitative insight into the subjective experiences of adherers to an exercise referral scheme: a thematic analysis. J Health Psychol.

[39] Shepperd JA, Rothman AJ, Klein WMP (2011). Using self- and identity-regulation to promote health: promises and challenges. Self Identity.

[40] Teixeira PJ, Carraça EV, Markland D, Silva MN, Ryan RM (2012). Exercise, physical activity, and self-determination theory: a systematic review. Int J Behav Nutr Phys Act.

[41] Sagsveen E, Rise MB, Grønning K, Westerlund H, Bratås O (2019). Respect, trust and continuity: a qualitative study exploring service users’ experience of involvement at a healthy life Centre in Norway. Health Expect.

[42] Ryan RM, Deci EL (2017). Self-determination theory: basic psychological needs in motivation, development, and wellness.

[43] Conradt M, Dierk JM, Schlumberger P, Rauh E, Hebebrand J, Rief W (2008). Who copes well? Obesity-related coping and its associations with shame, guilt, and weight loss. J Clin Psychol.

[44] Williams GC, Niemiec CP (2012). Positive affect and self-affirmation are beneficial, but do they facilitate maintenance of health-behavior change? A self-determination theory perspective. Arch Intern Med.

[45] Samdal GB, Eide GE, Barth T, Williams G, Meland E (2017). Effective behaviour change techniques for physical activity and healthy eating in overweight and obese adults: systematic review and meta-regression analyses. Int J Behav Nutr Phys Act.

[46] Hennessy EA, Johnson BT, Acabchuk RL, McCloskey K, Stewart-James J (2020). Self-regulation mechanisms in health behavior change: a systematic meta-review of meta-analyses, 2006-2017. Health Psychol Rev.

[47] Niemiec CP, Ryan RM, Deci EL, Williams GC (2009). Aspiring to physical health: the role of aspirations for physical health in facilitating long-term tobacco abstinence. Patient Educ Couns.

[48] Dennison RA, Ward RJ, Griffin SJ, Usher-Smith JA (2019). Women's views on lifestyle changes to reduce the risk of developing type 2 diabetes after gestational diabetes: a systematic review, qualitative synthesis and recommendations for practice. Diabet Med.

[49] Rothman AJ (2000). Toward a theory-based analysis of behavioral maintenance. Health Psychol.

[50] Blom EE, Aadland E, Skrove GK, Solbraa AK, Oldervoll LM (2019). Health-related quality of life and intensity-specific physical activity in high-risk adults attending a behavior change service within primary care. PLoS One.

[51] Cope DG (2014). Methods and meanings: credibility and trustworthiness of qualitative research. Oncol Nurs Forum.

